# Comment on Cifuentes-Suazo et al. Dietary Counseling: An Option to Malnutrition and Masticatory Deficiency in Patients with Total Protheses? A Scoping Review. *Nutrients* 2025, *17*, 141

**DOI:** 10.3390/nu18060944

**Published:** 2026-03-17

**Authors:** Héctor Fuentes-Barría, Raúl Aguilera-Eguía

**Affiliations:** 1 Centro de Investigación en Medicina de Altura (CEIMA), Universidad Arturo Prat, Iquique 1110939, Chile; 2 Departamento de Salud Pública, Facultad de Medicina, Universidad Católica de la Santísima Concepción, Concepción 3349001, Chile; raguilerae@ucsc.cl

Dear Editors,

We have carefully reviewed the article by Cifuentes-Suazo et al. [1] and would like to highlight several aspects essential to strengthen the rigor, transparency, and reproducibility of the review.

## 1. Typographical and Reporting Errors

We identified inconsistencies affecting clarity and reliability. The abbreviation *CRP* (Complete Removable Prostheses) is used inconsistently, contravening international recommendations for abbreviations [2]. Similarly, the acronym *CPA* appears in the Results section without being defined. Although likely a typographical error, its presence in the results cannot be considered a mere oversight, as it compromises manuscript credibility.

In the PRISMA flow diagram, “*Reports not retrieved*” is misused. According to PRISMA 2020 [3], this category applies only to articles that could not be retrieved, not to studies excluded for eligibility. This mislabeling undermines comparability and reproducibility and contradicts PRISMA’s transparency principles.

Additionally, inconsistencies between Figure 3 and the references cited in the discussion (references #26 and #29) create further confusion.

## 2. Methodological Concerns

Although described as a scoping review, inclusion was limited exclusively to randomized controlled trials (RCTs). While RCTs are the gold standard for evaluating interventions, PRISMA-ScR requires explicit justification for such restrictions [4]. The primary aim of a scoping review is to map available evidence, not to limit inclusion to a single study design [5,6]. Restricting RCTs undermines the exploratory nature of the methodology; the work should have been reported as a systematic review of effectiveness rather than a scoping review.

Similarly, the *Population–Concept–Context* (PCC) framework, essential in scoping reviews, is not described. PRISMA-ScR mandates explicit PCC reporting to ensure reproducibility and providing it only “by contacting the authors” does not replace reporting methods in the published article.

Excluding grey literature and not contacting authors critically limits the search’s comprehensiveness. Justifying this by “scope and feasibility” reflects convenience bias and contradicts international guidelines [5,6]. Furthermore, the study selection process omits key details, such as the number of reviewers, discrepancy resolution, or the use of software (e.g., RevMan, Rayyan), reducing reproducibility.

Regarding risk of bias, ROB2 and Robvis are mentioned only in Results, with no methodological description in Methods. Finally, the discussion establishes a relationship between nutrient intake and cognitive health based solely on one external article (Puri et al.). This inference introduces bias and lacks methodological soundness, as a review cannot base conclusions or hypotheses on a single non-systematic study.
